# Human Umbilical Cord Blood Mononuclear Cells in a Double-Hit Model of Bronchopulmonary Dysplasia in Neonatal Mice

**DOI:** 10.1371/journal.pone.0074740

**Published:** 2013-09-19

**Authors:** Dominik Monz, Erol Tutdibi, Céline Mildau, Jie Shen, Mariz Kasoha, Matthias W. Laschke, Torge Roolfs, Andreas Schmiedl, Thomas Tschernig, Karen Bieback, Ludwig Gortner

**Affiliations:** 1 Department of Pediatrics and Neonatology, Saarland University, Homburg, Saar, Germany; 2 Department of Pediatrics, Daping Hospital, Third Military Medical University, Chongqing, P. R. China; 3 Institute for Clinical and Experimental Surgery, Saarland University, Homburg, Saar, Germany; 4 Institute of Functional and Applied Anatomy, Hannover Medical School, Hannover, Germany; 5 Institute of Anatomy and Cell Biology, Saarland University, Homburg, Saar, Germany; 6 Institute of Transfusion Medicine and Immunology, University of Heidelberg, Mannheim, Germany; Childrens Hospital Los Angeles, United States of America

## Abstract

**Background:**

Bronchopulmonary dysplasia (BPD) presents a major threat of very preterm birth and treatment options are still limited. Stem cells from different sources have been used successfully in experimental BPD, induced by postnatal hyperoxia.

**Objectives:**

We investigated the effect of umbilical cord blood mononuclear cells (MNCs) in a new double-hit mouse model of BPD.

**Methods:**

For the double-hit, date mated mice were subjected to hypoxia and thereafter the offspring was exposed to hyperoxia. Human umbilical cord blood MNCs were given intraperitoneally by day P7. As outcome variables were defined: physical development (auxology), lung structure (histomorphometry), expression of markers for lung maturation and inflammation on mRNA and protein level. Pre- and postnatal normoxic pups and sham treated double-hit pups served as control groups.

**Results:**

Compared to normoxic controls, sham treated double-hit animals showed impaired physical and lung development with reduced alveolarization and increased thickness of septa. Electron microscopy revealed reduced volume density of lamellar bodies. Pulmonary expression of mRNA for surfactant proteins B and C, *Mtor* and *Crabp1* was reduced. Expression of *Igf1* was increased. Treatment with umbilical cord blood MNCs normalized thickness of septa and mRNA expression of *Mtor* to levels of normoxic controls. *Tgfb3* mRNA expression and pro-inflammatory IL-1β protein concentration were decreased.

**Conclusion:**

The results of our study demonstrate the therapeutic potential of umbilical cord blood MNCs in a new double-hit model of BPD in newborn mice. We found improved lung structure and effects on molecular level. Further studies are needed to address the role of systemic administration of MNCs in experimental BPD.

## Introduction

Bronchopulmonary dysplasia (BPD) is a major complication of extreme prematurity with a gestational age < 28 weeks [1]. Main pathogenetic features include alterations of lung growth and development with reduced alveolarization caused by prenatal hits such as intrauterine growth restriction (IUGR) and perinatal infections combined with postnatal ventilation and oxygen toxicity among others [2-4]. Thus, preterms with IUGR are at especially high risk of developing BPD [5]. Despite advances in therapy, BPD remains a major cause of neonatal morbidity and mortality [6]. Therapy of BPD is limited to moderately active drugs e.g. caffeine, and vitamin A or dexamethasone which has been shown to be associated with severe long term neuro-developmental adverse effects [7]. Furthermore, BPD itself carries a considerable risk for an impaired neuro-developmental prognosis and thus poses affected individuals at a considerable long term risk [8,9].

Recent data from stem cell therapies in various fields of medicine have described the potential for regeneration of impaired organ development [6,10]. Allogenic or xenogenic mesenchymal stem cells (MSCs) have been used in models of BPD induced by postnatal hyperoxia in neonatal rodents. Typical effects after either systemic or local pulmonary application of MSCs included an improved lung architecture compared to controls given sham treatment [11-14].

However, the duration until a standardized preparation of MSCs (involving cell isolation, expansion and characterization) can be provided may last more than 4 weeks [15]. As the pathogenesis of BPD starts already in utero and is sustained by postnatal stimuli, therapy should be started very early within the first week of life after birth [16]. Furthermore, due to the manufacturing process, MSCs are considered in Europe as Advanced Medicinal Product requiring a manufacturing license.

Umbilical cord blood is a rich source of different mononuclear cell populations containing high levels of primitive, multipotent stem cells, progenitor cells and immunoregulatory T cells among others [17]. For hematopoietic transplantation cord blood units are typically only volume reduced without further manipulation to obtain the mononuclear cell fraction. They can be easily processed within a short period of time and then stored either cryopreserved or injected as fresh unit. Umbilical cord blood MNCs are in clinical routine since years.

Therefore, we aimed to investigate the effects of human umbilical cord blood MNCs in a recently established double-hit mouse model of BPD combining pre- and postnatal noxious stimuli to the developing lung [2,5,6,18,19].

## Methods

Animal maintenance and the experiments were conducted in accordance with the European Communities Council Directive of 24 November 1986 (86/609/EEC) and were approved by the local board for animal welfare (Landesamt für Gesundheit und Verbraucherschutz Abteilung Lebensmittel und Veterinärwesen, Saarbrücken, Germany, AZ: C1 2.4.2.2). Conduct of the animal experiments followed the ARRIVE guidelines whenever possible [20]. The use of the human umbilical cord blood MNCs was approved by the ethics review committee of the responsible medical council (Ärztekammer des Saarlandes, Kenn. -Nr: 193/10). Written informed consent from the donor or the next of kin was obtained for use of this sample in research.

### Double-hit model of BPD

**Figure 1:**
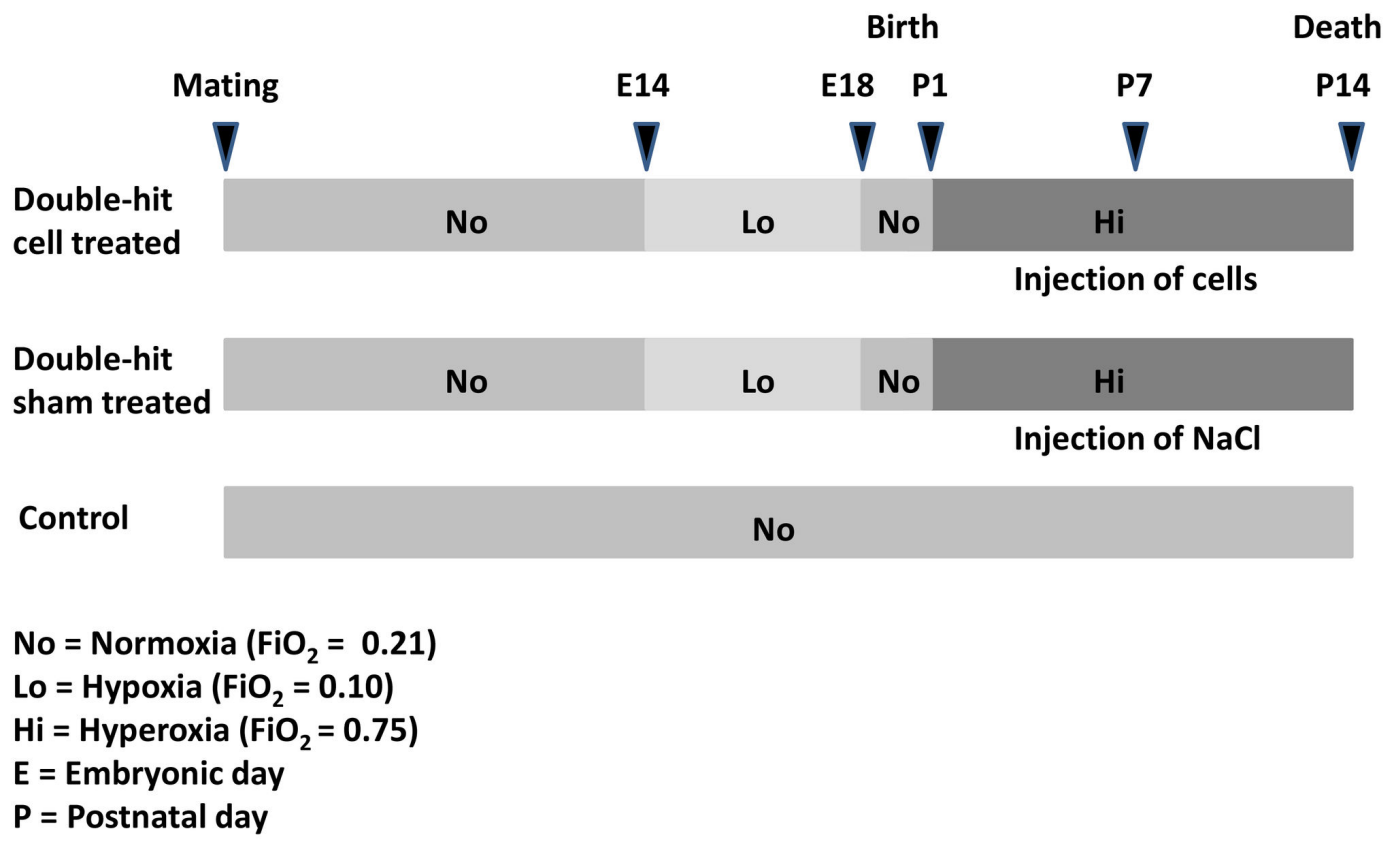
Schematic design of the experimental procedure. In the control group, the animals were constantly kept under normoxic conditions. In the double-hit groups, dams were exposed to hypoxia from day E14 to E18. After birth, the animals were kept in hyperoxia from day P1 to P14. The experimental double-hit group was given 2 x 10^5^ umbilical cord blood MNCs intraperitoneally. The double-hit control was sham treated, being given 0.9% NaCl solution.

We used a double-hit mouse model of BPD recently established by our group [19]. Date mated pregnant mice (C57BL/6N from Charles River, Sulzfeld, Germany) were housed in individual cages, being fed ad libitum. The dams were randomly assigned to a control (normoxia) or a double-hit group (Figure 1). In the double-hit group the pregnant dams were exposed to hypoxia (fraction of inspired oxygen (FiO_2_) 0.1) from gestational day 14 (E14) until day E18 to induce fetal growth restriction. After birth, the dams and their pups were exposed to hyperoxia (FiO_2_ 0.75) beginning from postnatal day 1 (P1) to day P14 to induce lung injury. Hypoxia and hyperoxia were induced in a ventilated chamber as described before [19]. Litters in the double-hit group were then randomly assigned to a sham treated and a MNC treated group. The administration of umbilical cord blood MNCs was performed at postnatal day P7 by intraperitoneal injection of a cell suspension containing 2 x 10^5^ cells in 0.9% NaCl. The animals in the sham treated group were administered the same volume of NaCl solution without cells. According to Heimfeld in stem cell transplantation median doses of CD34^+^ cells are in a magnitude of 3 x 10^6^/kg body weight, corresponding to 3 x 10^3^ cells/g body weight for the mouse [21]. We therefore chose a cell number of 2 x 10^5^ cells for the double-hit pups which had a median weight of approximately 3 grams at age P7. The normoxia control group comprised 3 dams and 22 pups. The double-hit group comprised 11 dams in total. Twenty-four pups received umbilical cord blood MNC treatment and 32 pups served as sham treated double-hit controls.

### Preparation of umbilical cord blood MNCs

Venous human umbilical cord blood was taken from healthy term neonates into a blood bag system (Stemcare CB COLLECT™, Fresenius Kabi, Bad Homburg, Germany) and stored at room temperature until further processing within 24 hours [22]. The cells were separated using Biocoll separation solution (density 1.077 g/ml, isotone by Biochrom AG, Berlin, Germany). Plasma and erythrocytes were discarded, cell numbers of the volume reduced MNCs were determined using a Coulter Ac·T diff cell counter. The cells were diluted in 0.9% NaCl and applied immediately at a dose of 2 x 10^5^.

### Auxological measurements

The body and lung weight of the pups were determined by means of a precision scale (Analytical Plus, Ohaus Europe, Switzerland) at P14. In addition, body length and fronto-occipital head length were measured using a digital caliper.

### Lung tissue preparation

The pups were sacrificed at P14 by injecting 400 mg pentobarbital per kg body weight intraperitoneally. The lungs were immediately removed, shock frozen in liquid nitrogen and stored at -80 °C for molecular analysis or fixed by tracheal instillation for morphological evaluation (0.1 M cacodylate buffer, 1.0% paraformaldehyde, 1.0% glutaraldehyde, pH 7.3 to 7.4) as described before [19].

### Stereological evaluation of lung tissue

The sampling and stereological methods were done according to the guidelines for quantitative assessment of lung structure [23].

The right single lung was embedded in 2% aqueous agar-agar and cut from apical to caudal into parallel slices. Starting with a random number, slices were alternatively taken for light and electron microscopy as described before [19]. The further processing of specimens was carried out as described [24] by rinsing tissue blocks repeatedly in 0.1 M cacodylate buffer, osmicating for 2 h in 1% OsO_4_ in 0.1 M cacodylate buffer and rinsing again in 0.1 M cacodylate buffer repeatedly (six changes during 60 min). After washing twice in distilled water, specimens were stained en bloc overnight (12-18 h) at 4-8 °C with a mixture of equal portions of uranyl-acetate and water (half-saturated aqueous uranyl-acetate solution (1:1)). After dehydration through an ascending series of alcohols (70%, 96%, 100%) whole tissue slices were embedded in a methacrylate resin (Technovit 7100; Kulzer, Heraeus, Germany). Sections of 1.5 µm thickness for quantitative light microscopic studies were stained with hematoxylin and eosin. For ultrastructural analysis specimens were dehydrated in an ascending series of acetone (70%, 90%, and 100%) and embedded in araldite and ultra-thin sections (70 nm) were cut and stained with lead citrate and uralyl-acetate in an ultrostainer (Leica, Bensheim, Germany).

Qualitative and stereological analyses were carried out on light as well as on electron microscopic level. Light microscopic analysis and gathering of alveolar epithelial cells type II (AEII) were performed as described before [19].

### Cytokine analysis

Cell lysate of pulmonary tissue was prepared and cytokine analysis was performed as described before on a Luminex 100, using the Exponent 3.2 software and Milliplex™ MAP for Luminex xMAP Technology Assays (MCYTOMAG-70K) by Millipore for the following analytes: IL-1β, IL-2, IL-10, IL-6, TNF-α, VEGF [19,25]. The standard concentrations ranged from 3.2 to 10,000 pg/ml.

### Lung mRNA isolation and gene expression analysis

Expression analysis was performed by following the MIQE guidelines wherever possible [26]. Total RNA was isolated and converted to cDNA as described earlier [19]. It was confirmed that the PCR results were not influenced by contaminating DNA in the RNA samples. The PCR efficiency and quality of the endogenous control was performed as described before [19]. PCR was performed using TaqMan^®^ Gene Expression Assays by Life Technologies (Table 1) on an Applied Biosystems 7500 Fast Real-Time PCR System with the 7500 Software v.2.0.5 (Life Technologies GmbH, Darmstadt, Germany). Relative quantification to the normoxia control group and normalization with the endogenous control were performed using the REST 2009 software (Qiagen, Hilden, Germany) with mean PCR efficiencies calculated from raw fluorescence data by linear regression using the LinRegPCR analysis software [27].

**Table 1:** TaqMan^®^ Gene Expression Assays (Life Technologies) used for quantitative RT-PCR.

**Gene**	**Taqman assay ID**
Surfactant-associated protein C - *SftpC*	Mm00488144_m1
Surfactant-associated protein B - *SftpB*	Mm00455681_m1
Elastin - *Eln*	Mm00514670_m1
Mammalian target of rapamycin - *Mtor*	Mm0044968_m1
Hypoxia inducible factor 1, alpha - *Hif1a*	Mm00468869_m1
Insulin-like growth factor 1 - *Igf1*	Mm00439560_m1
Vascular endothelial growth factor - *Vegf*	Mm01281449_m1
Transforming growth factor, beta 1 - *Tgfb1*	Mm01178820_m1
Transforming growth factor, beta 2 - *Tgfb2*	Mm00436955_m1
Transforming growth factor, beta 3 - *Tgfb3*	Mm00436960_m1
Retinoic acid receptor, alpha - *Rara*	Mm01296312_m1
Retinoic acid receptor, beta - *Rarb*	Mm01319677_m1
Retinoic acid receptor, gamma - *Rarg*	Mm00441091_m1
Retinol binding protein 1 - *Rbp1*	Mm00441119_m1
Cellular retinoic acid binding protein 1 - *Crabp1*	Mm00442776_m1
Actin, beta - *ActB* *	Mm00607939_s1

* endogenous control.

### Statistical analysis

Statistical analysis of the data describing the physical development of the mice and the bead measurement data was performed using IBM SPSS Statistics 19. Distribution was tested using the Kolmogorov-Smirnov and the Shapiro-Wilk tests. In case of a normal distribution the Student’s t-test was used to further analyze the data. If data were not normally distributed, they were analyzed using the Mann-Whitney-U test. Data are expressed as mean ± standard error of the mean (SEM) or as median and quartiles. For statistical analysis of gene expression data the REST 2009 tool (Qiagen, Hilden, Germany) was used [28]. Significance was assumed at p-values < 0.05.

## Results

### Auxological data

Growth variables of pups collected for each study group at P14 are given in table 2. Double-hit pups presented irrespective of treatment with MNCs the same impaired development with approximately 20% difference in growth compared to normoxic controls.

**Table 2:** Growth variables (as median and quartiles) of mouse pups at postnatal day P14.

	**Normoxic controls**	**Sham treated double-hit**	**Cell treated double-hit**
**Number of animals (lungs)**	22 (n = 11)	21 (n = 10)	20 (n = 9)
**Body weight [g**]	5.5 (5.3-7.7)	4.3 (3.9-4.6)	4.4 (3.9-4.8)
**Body length [mm**]	44.9 (41.8-47.2)	34.6 (31.4-35.4)	35.9 (34.1-37.3)
**Head length [mm**]	18.9 (18.6-19.6)	17.7 (17.2-20.1)	18 (17.8-19.6)
**Lung weight [g**]	0.09 (0.09-0.1)	0.07 (0.06-0.08)	0.07 (0.07-0.08)

Body weight, body length and lung weight showed significant changes between the normoxic control and both double-hit groups (p < 0.001 each). Lung weight was not determined in all animals (numbers for each group given in parenthesis).

**Table 3:** Cytokine concentrations (as median and quartiles) in lung tissue lysate of mouse pups at postnatal day P14.

	**Normoxic controls**	**Sham treated double-hit**	**Cell treated double-hit**
VEGF [pg/ml]	67.5 (51.8-94.7)	57.2 (42.8-79.8)	62.2 (34.4-80.6)
TNF-α [pg/ml]	< 3.2	< 3.2	< 3.2
IL-1β [pg/ml]#	2.6 (2.3-3.0)	1.6 (1.2-2.2)	2.8 (2.2-3.2)
IL-2 [pg/ml]	2.0 (1.9-2.2)	2.2 (2.2-2.5)	2.6 (2.2-4.6)
IL-6 [pg/ml]	< 3.2	< 3.2	< 3.2
IL-10 [pg/ml]	< 3.2	< 3.2	< 3.2

There were no significant differences between control and intervention groups, apart from IL-1β which was decreased in the sham treated double-hit group compared to the control group and the cell treated animals (p = 0.001 and 0.024 respectively). #significant change.

### Lung morphology

**Figure 2:**
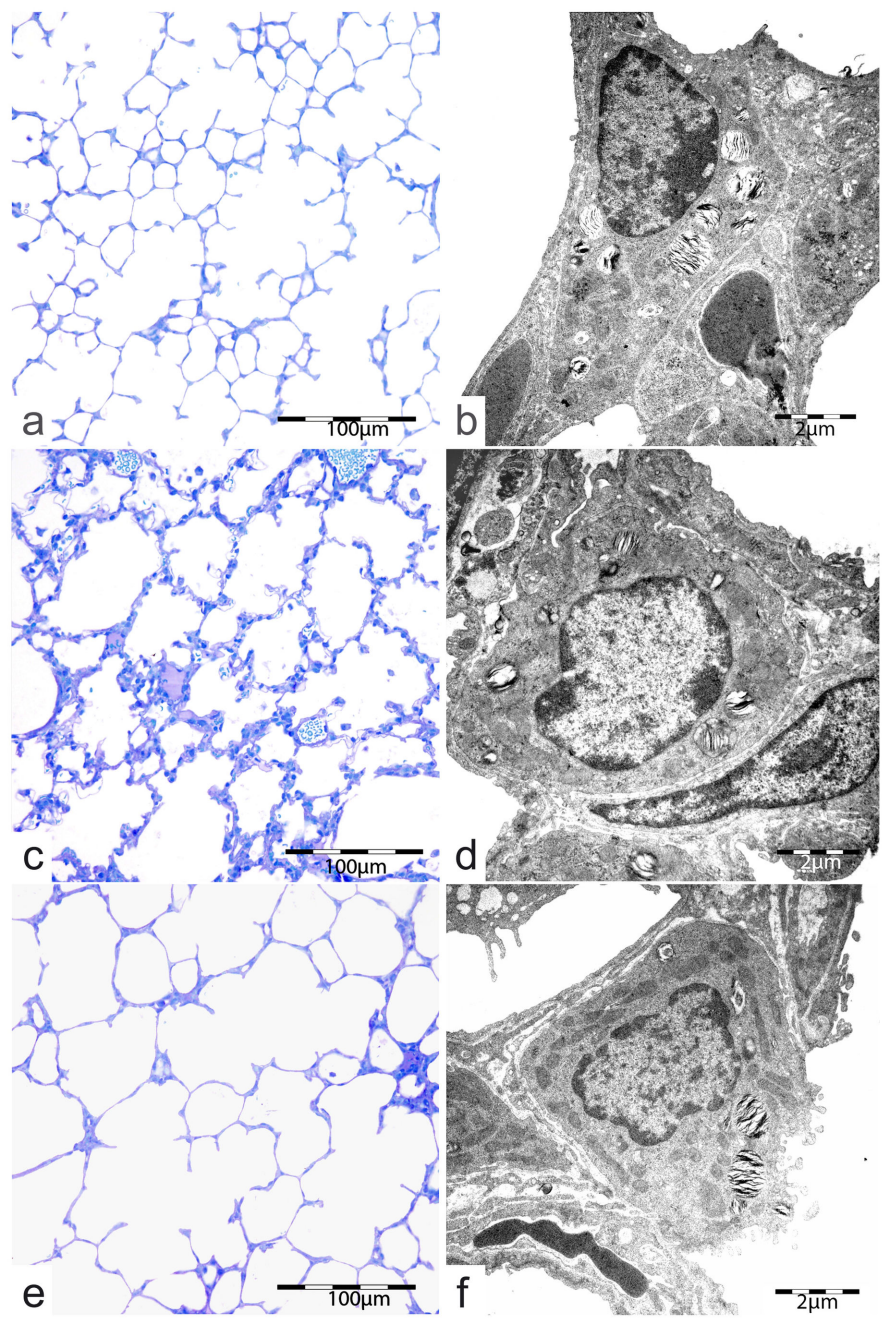
Pulmonary parenchyma and alveolar epithelial cells type II (AE II) of 14 days old controls (a, b), double-hit sham treated (c,d) and double-hit cell treated animals (e, f).

**Figure 3:**
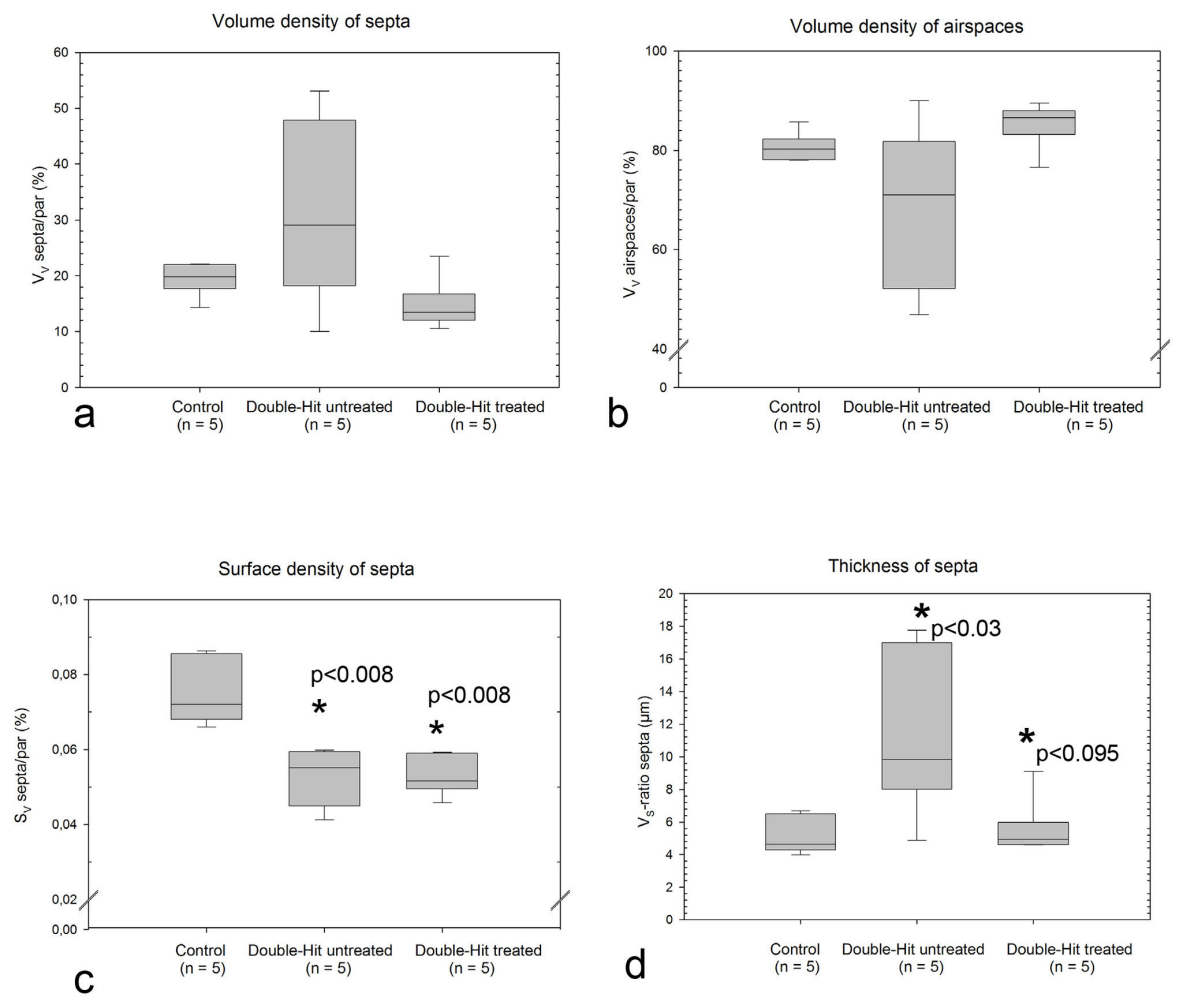
Stereological variables used for the characterization of lung parenchyma and its morphological maturity. a) Boxblots of the volume density of septa serving as an indirect measure for the development of sacculi and or alveoli. b) Boxplots of the volume density of air spaces serving as an indirect measure for the enfolding of air spaces and the degree of septal thickness. c) Boxplots of the surface density of septa serving as an indirect measure for alveolarization. The higher the septal surface density the higher is the formation of secondary septa as sign for alveolarization. P values obtained by the Mann-Whitney-Rank Sum Test. Significant differences compared to controls are stated. d) Boxplots of the thickness of septa as a measure for the morphological septal maturity. Decrease of the mean barrier septal thickness goes along with increased differentiation of septa. P values obtained by the Mann-Whitney-Rank Sum Test. P< 0.03 compared to controls, p<0.095 compared to the sham treated double-hit.

Figure 2 a-f show exemplary changes in lung architecture of normoxic controls and double-hit sham or MNC treated animals. Stereological evaluation was performed for quantification of pulmonary effects of MNCs. It could be shown that the lungs of the sham treated double-hit animals had a significantly higher septal barrier and lower septal surface density (Figure 3 a-d). Intraperitoneal injection of umbilical cord blood MNCs led to a relation of septa to air spaces which resembled to adequately developed normoxic control lungs (Figure 2 e). After MNC treatment the septa were significantly smaller than in the sham treated double-hit group and were comparable with the normoxic controls. However, the airspaces looked very wide in some areas. The reduced surface density of septa as an indirect variable for septal regeneration in sham treated double-hit pups was not influenced by MNC administration. Furthermore, the volume to surface density of airspaces had the highest values in the umbilical cord MNC treated double-hit group (16.16 ± 0.99, n=5). However, there was no significant difference to the sham treated group (13.71 ± 2.55, n=5). The lowest values were seen in the normoxic control groups (10.77 ± 0.57, n= 5).

The volume density of storage organelles of intracellular surfactant (lamellar bodies, Figure 2 b, d) was reduced in sham treated double-hit lungs indicating impaired biochemical development. The intracellular surfactant was not improved by the umbilical cord blood MNC application. The intracellular volume densities of lamellar bodies of AEII showed no difference between the sham treated double-hit and the umbilical cord blood MNC treated group (9.55 ± 0.52% vs. 9.27 ± 0.44%).

**Table 4:** Relative expression of different genes expressed in lungs of mouse pups at postnatal day P14.

**Gene**	Relative Expression in Double-hit untreated*	**p value**	**Regulation**	Relative Expression in Double-hit treated*	**p value**	**Regulation**
*Sftpc#*	0.528	0.005	down	0.653	0.014	down
*Sftpb#*	0.537	0.030	down	0.471	0.004	down
*Eln*	0.735	0.296	n.d.	0.761	0.333	n.d.
*Mtor#*	0.627	0.008	down	0.746	0.077	n.d.
*Hif1a*	0.798	0.337	n.d.	0.677	0.054	n.d.
*Igf1#*	2.06	0.001	up	2.043	<0.001	up
*Vegf*	0.762	0.206	n.d.	0.783	0.236	n.d.
*Tgfb1*	1.084	0.718	n.d.	1.143	0.541	n.d.
*Tgfb2*	1.417	0.159	n.d.	1.473	0.127	n.d.
*Tgfb3#*	0.765	0.135	n.d.	0.527	0.003	down
*Rara*	0.521	0.117	n.d.	0.693	0.374	n.d.
*Rarb*	0.998	0.994	n.d.	1.293	0.270	n.d.
*Rarg*	0.620	0.198	n.d.	0.679	0.252	n.d.
*Rbp1*	0.826	0.438	n.d.	0.919	0.726	n.d.
*Crabp1#*	0.026	0.026	down	0.023	<0.001	down

* Relative expression was determined in relation to the expression in lungs of control normoxic mice set at 1, using the ΔΔCt-method implemented in the REST 2009 software (Qiagen, Hilden, Germany). Up: up-regulated, down, down-regulated, n.d. no difference. #Genes with significantly changed relative expression.

### Lung tissue cytokine levels

The concentrations of IL-1β, IL-2, IL-10, IL-6, TNF-α, VEGF taken from lung tissue are given in table 3. Only IL-1β was decreased in the sham treated double-hit group compared to the control group and the MNC treated animals (p = 0.001 and 0.024 respectively).

### Gene expression

Relative expression data of above mentioned genes (Table 1) analyzed in this study is given in table 4. In the sham treated double-hit group *SftpC*, *SftpB*, C*rabp1* and *Mtor* were down-regulated, and *Igf1* was up-regulated in comparison to normoxic controls. In the cell treated double-hit group *Tgfb3* was significantly down-regulated. *Mtor* expression showed no difference between the MNC treated double-hit and the normoxic control group.

## Discussion

The main goal of this study was to investigate the effect of umbilical cord blood cells in a double-hit model of BPD according to the following criteria:

- physical development by auxologic measurements,
- lung development on light and electron microscopic evaluation,
- expression of markers for lung maturation or inflammation.

Sham treated and umbilical cord blood MNC treated double-hit animals were smaller and less developed than normoxic controls. Treatment with umbilical cord blood MNCs did not result in improved physical development compared to sham treated animals.

In double-hit mice alveolar septa in the lung are thicker compared to the lung of normally developed mice [19]. After treatment with umbilical cord blood MNCs the size of the septa was the same as in normally developed lungs. These findings indicate an improvement in lung architecture after treatment with umbilical cord blood MNCs.

Analyses on electron microscopic level showed a reduced subcellular volume density of lamellar bodies in AEII in double-hit mice which was not influenced by umbilical cord blood MNC treatment. As lamellar bodies are the intracellular units of surfactant storage, the reduced number of these organelles indicates reduced surfactant. These histomorphometric findings are supported by expression analyses.

As a marker for functional development of the lung the regulation of surfactant protein encoding genes was analyzed by measuring *Sftpb* and *Sftpc* expression which was clearly reduced in the double-hit model in comparison to the lung of normally developed mice. Treatment with umbilical cord blood MNCs did not have any effect on this down-regulation.

*Igf1* expression was up-regulated in the same range in lungs from both double-hit groups. The *Igf1* up-regulation in experimental BPD is in accordance with findings of other groups, describing elevated IGF1 in children affected by BPD (Chetty et al, 2004, Capoluongo et al 2007).

The transcription of the gene for the cellular retinoic acid binding protein 1, *Crabp1*, was down-regulated in both double-hit groups compared to normal lungs.

In sham and MNC treated double-hit animals *Tgfb1* and *Tgfb2* expression in the lung did not differ from expression in normoxic control animals. In premature neonates with BPD TGF-β is increased in the bronchoalveolar lavage [29]. Conditional overexpression of TGF-β1 in the lungs of neonatal mice and rats results in interstitial fibrosis that is comparable with BPD in humans [30,31], *Tgfb3* was on the same level as in normoxic animals in the sham treated animals, but was down-regulated in lung tissue of the MNC treated animals. TGF-β signalling plays a crucial role during lung development and increased TGF-β levels negatively affect alveogenesis [29,32]. On the other hand, impairment of alveolarization was shown in mice after Smad3, a member of the protein family responsible for intracellular TGF-β signalling was knocked out [33]. Early during alveolarization, increased TGF-β inhibits further development of alveoli while it causes pulmonary fibrosis in lungs with advanced alveolarization [32,33]. These findings may indicate that adequate TGF-β signalling plays a crucial role in alveolarization. Not only overexpression of TGF-β, but an impaired TGF-β pathway in general has an impact on lung development.

In our study, we were not able to find increased expression of the genes for the TGF-β family *Tgfb1*, *Tgfb2*, and *Tgfb3* in the sham-treated control group compared to the normoxic controls. Interestingly, *Tgfb3* was down-regulated after treatment with umbilical cord blood MNCs, while *Tgfb1* and *Tgfb2* expression did not show any changes. The overall level of TGF-β was thus reduced. Still, the exact meaning of our findings remains unclear at the moment.

Interestingly, we found a significant change in *Mtor* expression after umbilical cord blood MNC treatment compared with controls. The protein mTOR represents a kinase which is involved in key functions promoting cell growth and metabolism [34,35]. Mammalian TOR (mTOR) is ubiquitously expressed in tissue and is activated by growth factors, nutrients, oxygen, energy and inflammation. Deregulation of mTOR activity is linked to a variety of human diseases including cancer, obesity, type 2 diabetes, and neurodegeneration [34,36,37].

In our double-hit model the expression of *Mtor* was clearly reduced in the sham treated animals. Umbilical cord blood MNC treatment limited the reduction of *Mtor* expression. *Mtor* expression showed no difference between the MNC treated double-hit and the normoxic control group. Thus, umbilical cord blood MNCs may promote cell growth and with that lung development by up-regulating *Mtor* expression.

There is emerging evidence for the mTOR signalling network playing an important role in a number of processes critical for development of pulmonary vascular remodelling in pulmonary hypertension and pulmonary arterial hypertension [38]. Fielhaber and co-workers demonstrated that inhibition of mTOR enhanced lung injury and cellular apoptosis in mice exposed to LPS suggesting that mTOR suppresses proapoptotic responses and lung injury [39].

We were able to show that the use of umbilical cord blood MNCs in our double-hit model of BPD in mice had a regulatory effect on the expression of *Mtor* in the lung.

Elevated serum concentrations of pro-inflammatory cytokines IL-1β, IL-6, IL-8 and the anti-inflammatory IL-10 are markers of the pathogenesis of BPD in extremely low birth weight neonates [40]. In contrast to this, we found IL-1β to be less expressed in sham treated double-hit animals compared to normal and umbilical cord blood MNC treated animals in our model. It was shown by Bry and co-workers that overexpression of IL-1β alone can induce a BPD-like disorder in neonatal mice [40]. We found decreased IL-1β concentrations in the lungs of our sham treated double-hit mice, while IL-6 and IL-10 did not reveal any changes between the different experimental groups, indicating that processes independent of IL-1β elevation also play an important role in the pathogenesis of BPD. This is in accordance with the fact that new type BPD commonly shows less pronounced signs of inflammation compared to the old type [41,42].

Our rationale for using a “crude” cell preparation from umbilical cord blood was that it is easily and quickly available for early treatment of BPD and that there is no further manipulation of the cells needed before transplantation. Early treatment at postnatal day 3 (P3) with mesenchymal stem cells (MSCs) in the classical BPD model showed significant protection compared to late treatment at day P10 [16].

Using umbilical cord blood MNCs in our double-hit model of BPD in mice, we were not able to see such a strong protective effect as observed with MSC preparations [11,13,14,43]. This may be explained by several reasons: 1) Our “crude” cell preparations were too heterogeneous to give a clear effect, while pure MSC preparations were able to do so. Nevertheless, MNCs have particular advantages regarding their fast and easy availability and proven biological activities. 2) The timing of our treatment at day P7 was too late. Lung structure may have been too much impaired for the cells to have adequate impact. 3) The nature of our model combining prenatal hypoxia with postnatal hyperoxia. Prenatal hypoxia on its own already affects expression of *Sftpa*, *Sftpb* and *Sftpc* besides inducing growth restriction [18]. It was also shown in lambs that growth restriction has a negative effect on lung development and function [44]. Furthermore an adverse prenatal environment may have developmental effects and influence postnatal health without being obvious at birth or near birth [45-48]. Thus, the combination of prenatal and postnatal hits affecting normal lung development, as well as cell preparation and timing of application may have attenuated the strong effect seen with stem cells in the classic animal model of BPD based on only the postnatal hit.

To our best knowledge this is the first study about the translational potential of umbilical cord blood MNCs in a model of BPD, combining a prenatal hit inducing growth restriction with a postnatal hit inducing lung damage.

In conclusion we found that treatment with umbilical cord blood MNCs did not have as strong an effect in a double-hit mouse model of BPD as do pure MSCs in the classic model. Further studies to improve the effect are needed. Nonetheless umbilical cord blood MNCs give prospect to regenerative therapy of BPD using autologous cells without further manipulation during the first days following birth.
